# The Prevalence, Epidemiological, and Molecular Characterization of Methicillin-Resistant *Staphylococcus aureus* (MRSA) in Macau (2017–2022)

**DOI:** 10.3390/microorganisms12010148

**Published:** 2024-01-12

**Authors:** Abao Xing, Hoi Man Ng, Huining Jiao, Kefeng Li, Qianhong Ye

**Affiliations:** 1 Faculty of Applied Sciences, Macao Polytechnic University, Macau; p2314405@mpu.edu.mo (A.X.); kefengl@mpu.edu.mo (K.L.); 2 Clinical Laboratory, Kiang Wu Hospital, Macau; alvin_ng@163.com; 3 Faculty of Health Sciences and Sports, Macao Polytechnic University, Macau; p2010371@mpu.edu.mo

**Keywords:** Macau, methicillin-resistant *Staphylococcus aureus*, nosocomial infection, *SCCmec* typing, antimicrobial susceptibility

## Abstract

Macau, recognized as a global tourism hub and the world’s most densely populated region, provides a unique environment conducive to methicillin-resistant *Staphylococcus aureus* (MRSA) transmission in healthcare and community settings, posing a significant public health concern both locally and globally. The epidemiology and molecular characteristics of MRSA in the distinct city of Macau remain largely unelucidated. This five-year longitudinal study (2017–2022) examined the local prevalence and molecular typing of MRSA in Macau, with future MRSA type distribution predicted through ARIMA modeling. We subsequently analyzed the epidemiological characteristics of MRSA, including specimen source, clinical department, collection year, season, patient age, sex, and the annual number of tourists. Comprehensive antibiotic resistance profiles of the strains were also assessed. Of 504 clinically isolated *S. aureus* strains, 183 (36.3%) were identified as MRSA by the cefoxitin disk diffusion method and validated through multi-locus sequence typing (MLST). The MRSA detection rate showed an upward trend, increasing from 30.1% in 2017 to 45.7% in 2022. *SCCmec* type IV was predominant (28.9%), followed by types II (25.4%), III (22.1%), and V (22.1%). The primary sources of MRSA isolates were sputum (39.2%) and secretions (25.6%). Older age emerged as a risk factor for MRSA infection, whereas no significant associations were found with seasonal variations, gender, or the annual number of tourists. Despite displaying universal resistance to cefoxitin, oxacillin, and benzylpenicillin, MRSA isolates in Macau remained fully sensitive to vancomycin, tigecycline, quinupristin, nitrofurantoin, and linezolid. Continuous surveillance and analysis of MRSA distribution in Macau could provide invaluable insights for the effective management of MRSA prevention and control measures within healthcare settings.

## 1. Introduction

Methicillin-resistant *Staphylococcus aureus* (MRSA) strains have spread rapidly worldwide since the 1990s. The increasing prevalence of MRSA and continual development of drug resistance mechanisms have posed substantial challenges for the clinical management of infections [1,2]. MRSA is highly pathogenic, capable of causing severe progressive and necrotizing diseases, including bacteremia, sepsis, and toxic shock syndrome [3]. According to the monitoring report from the China Antimicrobial Surveillance Network (CHINET), the detection rate of *Staphylococcus aureus* (SA), particularly MRSA, ranks first among Gram-positive bacteria [4]. It is considered one of the most severe pathogenic bacteria in clinical settings. In recent years, with the widespread use of antibiotics such as methicillin, the resistance of SA, especially MRSA, has gradually increased, leading to a global prevalence of methicillin-resistant strains. Studies have demonstrated that, compared to methicillin-susceptible *Staphylococcus aureus* (MSSA), MRSA exhibits higher morbidity, mortality rates, and incurs elevated healthcare costs, posing a significant threat to global public health [5,6,7]. According to reports, in many Asian countries, the prevalence of MRSA in hospitals can reach as high as 50–60% [8,9]. In China, despite a recent decline in MRSA incidence, monitoring reports indicate an ongoing prevalence of over 30% annually [10]. MRSA infections rank second in attributable mortality among all drug-resistant bacteria and independently contribute to mortality in *S. aureus* bloodstream infections [11]. Moreover, due to continuous strain evolution, antibiotic iteration and updates, as well as increased migration frequencies among different regions, MRSA infections have become more complex and diverse, presenting new challenges to the management of MRSA infections. Consequently, continued surveillance and analysis of trends in MRSA prevalence are critically important. These efforts can elucidate patterns of resistance, inform treatment strategies, and guide the development of interventions to mitigate MRSA transmission.

Geographically isolated yet densely populated, Macau has several distinctive characteristics that warrant investigation of the local prevalence and molecular epidemiology of MRSA. Situated on the border of the tropics just 60 km from Hong Kong, Macau has the highest population density globally, with over 20,000 residents per square kilometer. Culturally, Macau retains substantial Portuguese influence after over 400 years of colonial rule. Many residents maintain European customs, and a proportion of doctors in local hospitals are foreign-trained. Moreover, most clinical pharmaceuticals are imported. As a tourism and gaming hub, Macau attracts a high volume of international travelers, which may facilitate MRSA transmission in healthcare and community settings [12,13]. Based on these factors, the prevalence of MRSA and the molecular epidemic characteristics of Macau MRSA may have its uniqueness compared to mainland China and Hong Kong.

High MRSA detection rates have been documented in mainland China, Europe, and Hong Kong. The 2021 China Antimicrobial Surveillance Network (CHINET) reported that 31.8% of clinically isolated pathogenic strains in China were *S. aureus*, with an MRSA detection rate of 29.4% among these isolates [14]. The European Antimicrobial Resistance Surveillance System (EARSS) noted that 22.1% of resistant strains isolated clinically in 2021 were MRSA [15]. In Hong Kong, methicillin resistance has exceeded 50% among *S. aureus* isolates in the last few years [16]. However, despite the high regional prevalence, the epidemiology and molecular characteristics of MRSA in the distinct city of Macau remain largely unelucidated.

Methicillin resistance is mediated by the *mecA* gene, located on the staphylococcal cassette chromosome mec (*SCCmec*), a mobile genetic element integrated into the bacterial chromosome [17,18,19]. *SCCmec* also harbors a set of site-specific recombinase genes (*ccrA* and *ccrB*) that are responsible for its mobility. Based on variations in the *mec* and *ccr* complexes, *SCCmec* elements are currently classified into types I to XIV as of December, 2021, with further division into subtypes by J region differences (e.g., IVa, IVb, and IVc) [20]. Previous studies indicated that healthcare-associated (HA) MRSA (HA-MRSA) infections often involve multidrug-resistant strains with *SCCmec* types I, II, or III, rarely type IV [21]. In contrast, *SCCmec* type IV predominates in community-associated MRSA (CA-MRSA) [22]. Additionally, MRSA strains were found to exhibit regional distinctions in drug resistance profiles and epidemic types, such as type III in Malaysia [23], type II in Israel [24] and Japan [25], and type III in China [26]. Therefore, characterizing the local *SCCmec* types is crucial to understanding MRSA epidemiology in a given region.

In this study, we investigated the prevalence of MRSA in clinical settings in Macau over the past 5 years, from 2017 to 2022. MRSA was isolated from clinical samples obtained at local hospitals during this period. We characterized the *SCCmec* types of the isolated MRSA strains and comprehensively analyzed their distribution, epidemiological features, and drug resistance profiles. Furthermore, we explored the associations between MRSA occurrence and age, sex, seasonal variation, and the annual number of travelers to Macau during the 5-year period.

## 2. Materials and Methods

Brief methods are summarized below, and the details of methodology can be found in the Supplementary Material/Methods section.

### 2.1. Sample Collection and Strain Isolation

Kiang Wu Hospital is one of the largest hospitals in Macau with a total of 736 beds and is equipped with 19 clinical departments, including internal medicine, surgery, obstetrics and gynecology, and laboratory medicine. In 2022, the emergency department treated 1.37 million patients, and there were 28,300 discharges (http://www.kwh.org.mo/kwh/, accessed on 1 December 2023). Samples in this study were selected from all patients who sought medical care at Kiang Wu Hospital, with an age range from 7 days to 101 years between September 2017 and April 2022. *S. aureus* isolates were recovered from inpatients and outpatients who had cough, fever, skin abscess, and other infection-related clinical symptoms using sterile swabs or other appropriate collection tools from sites relevant to *S. aureus* colonization or infection, such as nasal passages, wounds, or skin lesions. A total of 504 non-duplicate strains of *S. aureus* were collected and isolated from the Departments of Respiratory Medicine, General Surgery, Dermatology, Neurology, and others. Among them, 276 were males, 184 were females, and 47 cases had unrecorded gender information. Various sample types were collected from patients, including pus, urine, blood, sputum, secretions, and ascites. All strains were identified using the VITEK-2 automated microbial identification system (bioMérieux, Lyon, France). To confirm whether the isolated strains were MSSA or MRSA, multi-locus sequence typing (MLST) was conducted by randomly selected 2–3 isolates from each survey period. The study protocols were approved by the Institutional Review Board (IRB) of Kiang Wu Hospital (Protocol ID#: 2018-004).

### 2.2. SCCmec Typing

The *SCCmec* typing of MRSA isolates was performed using the multiplex PCR method [27]. All oligonucleotide primers used in this study were synthesized by Sangon Biotech (Shanghai, China). *SCCmec* types I–V were identified based on the combination of the cassette chromosome recombinase (ccr) type and mec class. The primers and PCR programs for amplification of the relevant genes were listed in Supplementary Table S1. After the reaction, a 6× DNA loading buffer was added to the PCR products, and 20 μL of the mixture was loaded onto a 1.2% agarose gel for electrophoresis at 110 V for 40 min. The gel was visualized, and images were captured using a UV gel imaging system. *S. aureus* ATCC43300, ATCC25923, ATCC33591, and ATCC49775 were used as quality control strains.

### 2.3. Multi-Locus Sequence Typing

To confirm whether the isolated strains were MSSA or MRSA, Multi-locus sequence typing (MLST) was conducted by randomly selecting 2–3 isolates from each survey period. According to the PubMLST database (https://pubmlst.org/multilocus-sequence-typing, accessed on 12 December 2023), perform PCR using the provided primer sequences and conditions (https://pubmlst.org/organisms/staphylococcus-aureus/primers, accessed on 12 December 2023) for the 7 housekeeping genes (*arcC*, *aroE*, *glpF*, *gmk*, *pta*, *tpi* and *yqiL*). Subsequently, submit the successfully amplified PCR products to Sangon Biotech (Shanghai, China) for bidirectional sequencing. After receiving the sequencing results, use DNASTAR for sequence assembly. Upload the assembled sequences to the typing online platform (https://pubmlst.org/bigsdb?db=pubmlst_saureus_seqdef, accessed on 12 December 2023) of PubMLST to retrieve allelic profiles and corresponding sequence types (STs) and clonal complexes (CCs).

### 2.4. Sequence Analysis

Amplified gel bands were excised under UV light using a cutter and purified with the Gene ALL Gel Purification Kit per the manufacturer’s protocol. Purified samples were sequenced by Sangon Biotech (Shanghai, China). The nucleotide sequences were aligned with Clustal W using the msa R package (v1.30.1). A phylogenetic tree was then constructed using neighbor-joining (NJ) methods using the ape (v5.7-1) R package and visualized by the ggtree (v3.6.2) package in R.

### 2.5. Epidemilogical Analysis

The occurrence of MRSA was stratified by specimen source, clinical department, collection year, season, patient age, and sex, and the differences between these factors were compared using Chi-square analysis. Tourism data on annual tourists in Macau from 2017 to 2022 were retrieved from the Macao Government Tourism Office (https://dataplus.macaotourism.gov.mo/, accessed on 21 October 2023). The associations between MRSA occurrence and age and the annual number of international travelers were analyzed using Pearson’s correlation.

### 2.6. Autoregressive Integrated Moving Average (ARIMA) Modeling

The ARIMA modeling was employed to forecast future MRSA prevalence trends in Macau using the R packages, tseries (v0.10-53) and forecast (v8.21.1). The model was fitted to the past five years of MRSA typing data collected in this study from 2017 to 2022.

### 2.7. Antimicrobial Susceptibility Testing

All clinical specimens for isolation and cultivation were processed according to the National Operating Procedures for Clinical Laboratory Testing. Antimicrobial susceptibility of the 504 strains of *S. aureus* was tested against 16 drugs using VITEK-2 AST cards (bioMérieux, France) based on the manufacturer’s standard protocol. The tested antibiotics were: cefoxitin, benzylpenicillin, oxacillin, linezolid, gentamicin, ciprofloxacin, levofloxacin, moxifloxacin, erythromycin, clindamycin, quinupristin, vancomycin, tetracycline, tigecycline, nitrofurantoin, and rifampicin. The minimum inhibitory concentration (MIC) breakpoints of antimicrobials for *S. aureus* using VITEK-2 COMPACT system can be referred to in Supplementary Table S2. The criteria for antimicrobial susceptibility determination and quality control were based on the 2017 Clinical and Laboratory Standards Institute (CLSI) guidelines. The MRSA phenotype was determined using the cefoxitin disk diffusion method [28]. Further details can be found in the Supplementary Material/Methods section.

### 2.8. Statistical Analysis

All statistical analyses were performed in R (v4.2.1) unless otherwise specified. Continuous data are expressed as mean ± standard deviation (SD) or median and interquartile range (IQR) based on distribution. The categorical variables are shown as value (%). Chi-squared tests were performed for the comparison of categorical variables. Person’s correlation analysis was performed to analyze the associations between MRSA occurrence and the continuous variables. The proportions were compared using two-proportional z-tests. *p* < 0.05 was considered statistically significant. Data were visualized using R packages of ggplot2 (v3.4.2), seqinr (v4.2-30), pheatmap (v1.0.12), and ggtree.

## 3. Results

### 3.1. Overview of the Study Design

**Figure 1:**
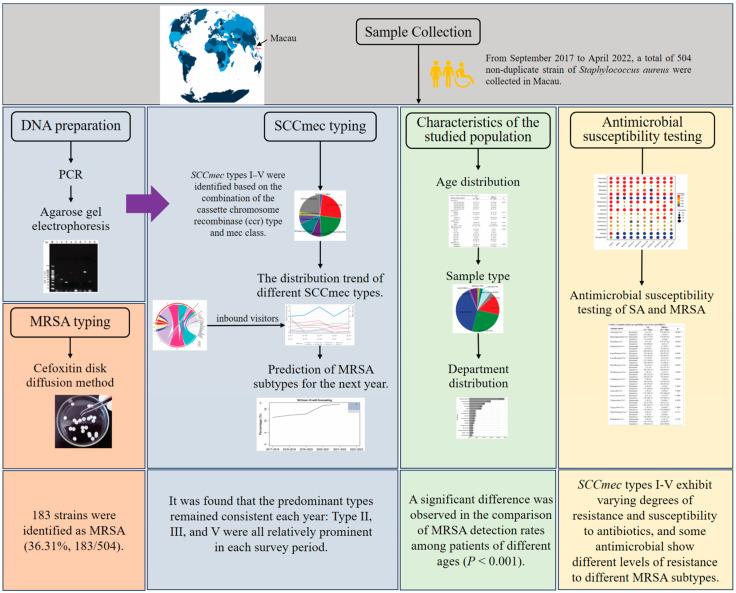
The overall design of this study.

The study design is outlined in Figure 1. During the period from 2017 to 2022, *S. aureus* strains were collected annually from September to the following April at Kiang Wu Hospital, the main hospital in Macau. MRSA identification was done by cefoxitin disk diffusion and further confirmed by VITEK-2 microbiology analysis. The main *SCCmec* types (I–V) were identified using multiplex PCR and sequencing. The epidemiological characteristics of MRSA in Macau were then analyzed, including specimen source, clinical department, collection year, season, patient age, and sex. Subsequently, the comprehensive antibiotic resistance profiles of Macau MRSA strains were explored.

### 3.2. SCCmec (I~V) Gene Detection

Electrophoresis and Nanodrop quantification showed high-quality bacterial DNA extracted from MRSA strains and quality control strains. Agarose gel electrophoresis of PCR products for the eight candidate reference genes displayed single bands at expected sizes (Figure S1A). The phylogenetic relationships of the 8 *SCCmec* genes were assessed by generating a phylogenetic tree based on DNA sequences (Figure S1B). Sequence alignment and a similarity heatmap demonstrated high sequence similarity among the 8 genes (Figure S1C,D).

### 3.3. Multi-Locus Sequence Typing of S. aureus Isolates

**Figure 2:**
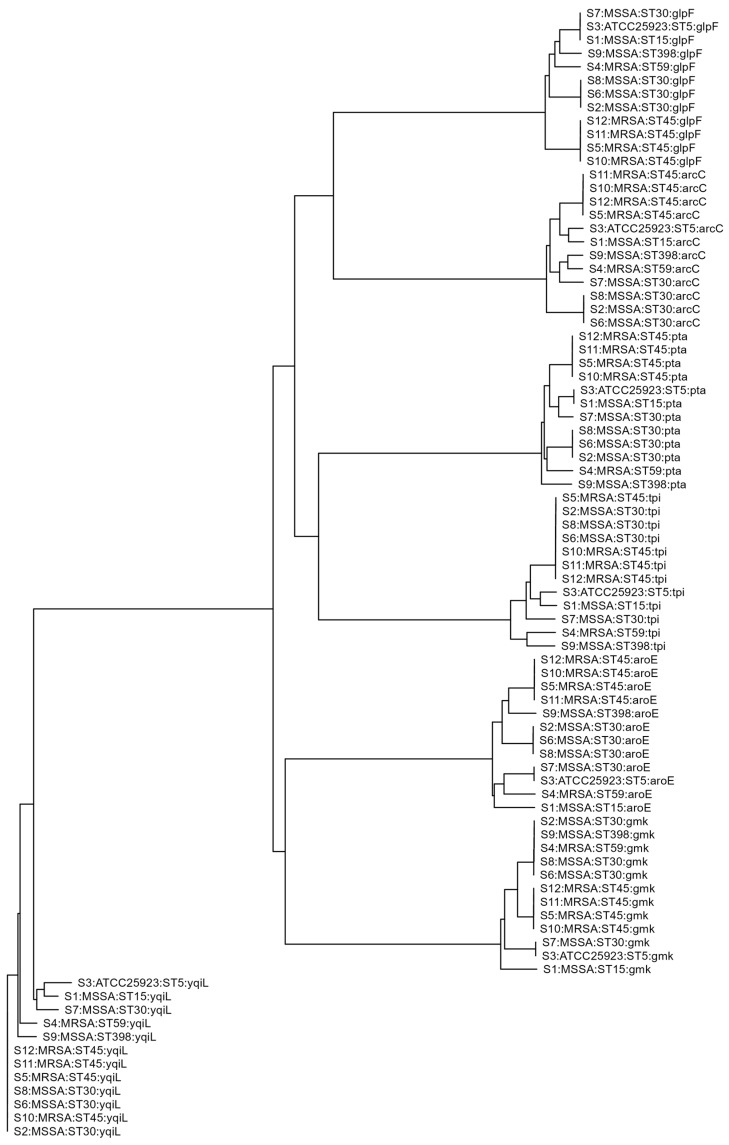
Dendrogram showing the genetic relatedness of the 12 *S. aureus* isolates. STs are labeled on the right. For example, S2: MSSA:ST30:yqiL, where S2 means Sample ID; MSSA means *S. aureus* type, methicillin-resistant or methicillin-susceptible; ST30 means multi-locus sequence type of SA; *yqiL* is one of the 7 housekeeping genes. MRSA: Methicillin-resistant *Staphylococcus aureus*; MSSA: Methicillin-susceptible *Staphylococcus aureus*.

The sequencing data were uploaded to the PubMLST database for online analysis. Each *S. aureus* isolate was assigned a sequence type (ST) and clonal complex (CC) based on the combination of 7 housekeeping genes, known as the allelic profile (Table 1). Previous studies [29,30,31,32,33,34] have confirmed that ST5, ST7, ST15, ST30, and ST398 are MSSA, while ST45 and ST59 are MRSA, which is consistent with the results obtained from the cefoxitin disk diffusion method for MRSA identification. Subsequently, a dendrogram was constructed to conduct evolutionary analysis and assess the genetic relatedness among these strains (Figure 2).

### 3.4. Molecular Typing of MRSA Strains in Macau

Using *SCCmec* typing, 8 types and subtypes (I, II, III, IVa, IVb, IVc, IVd, V) were identified among the 183 MRSA isolates. Comparison of typing by survey period (Figure S2A–E) revealed that the predominant MRSA type varied annually. However, from the figures, it can be observed that Type II, III, IV, and V are relatively more prevalent, with Type I constituting a relatively small proportion. The results from the five survey periods reflect inconsistent patterns of predominant types, indicating that the MRSA typing landscape in Macau is dynamically changing. Overall, type IV was the most predominant (28.8%), accounting for approximately one-sixth of all MRSA isolates (Figure S2F), followed by III (25.4%), III (22.0%), and V (22.0%). Two isolates were non-typable (NT) by *SCCmec*.

### 3.5. Epidemilogical Characteristics of MRSA Strains in Macau

During the study period from 2017 to 2022, a total of 504 strains of *S. aureus* were identified (Table 2), of which 183 were MRSA (36.3%) and 321 were MSSA (63.7%). The highest number of MRSA cases was detected in 2021–2022 (58 strains), with the highest detection rate of MRSA in 2019–2020 (47.9%, 23/48). The MRSA detection rates of other years were 30.1% (2017–2018), 33.3% (2018–2019), 30.0% (2020–2021), and 45.7% (2021–2022), respectively. Chi-square analysis revealed significant differences in MRSA detection rates between years (*p* = 0.018) and in MRSA detection rates between different years (Table 2). Due to the peak tourism season in Macau during the Winter and Spring, MRSA occurrence tends to be relatively higher during this period, but seasonal differences were not significant (*p* = 0.13), as shown in Table 2.

No statistical significance was found between MRSA infection and gender (*p* = 0.36). However, MRSA detection rates significantly differed by age (*p* < 0.001). As shown in Table 2, the average age of the susceptible cases was 47.2 ± 31.9, which was lower than the average age of the MRSA case (62.2 ± 28.4), suggesting increased MRSA susceptibility among older patients. Additionally, outpatients showed lower MRSA detection (29.4%, 37/126) compared to inpatients (43.6%, 122/280) (*p* = 0.009, Table 2).

Table 3 compares the molecular typing of MRSA infections between inpatient and outpatient groups. The data demonstrate that *SCCmec* III was significantly more prevalent among inpatients (8.9%) compared to the outpatient group (2.4%), with a significant difference of *p* = 0.028. Conversely, there are no statistically significant differences in the distribution of *SCCmec* I, II, IV, and V between inpatients and outpatients (*p* > 0.05). These findings suggest that *SCCmec* III MRSA may be more closely associated with nosocomial infections in Macau, whereas the other SCCmec types appear to play a relatively balanced role in the transmission of MRSA among both inpatient and outpatient settings.

**Figure 3:**
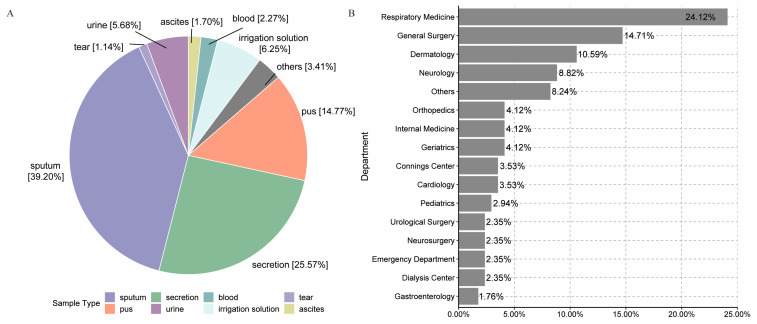
MRSA detection rates in different specimens (**A**) and clinical departments (**B**). Note: for specimen (**A**), others contain abdominal drainage tube, articular matter, synovial fluid, skin, endotracheal tube, and body fluid. For clinical departments (**B**), others include Breast Clinic, Cardiothoracic Surgery, Diabetes Prevention and Control Center, Endocrinology, Intensive Care Unit, Laboratory, Nephrology, Obstetrics and Gynecology, Oncology, Ophthalmology, Otorhinolaryngology, Outreach Service Clinic, Rheumatology and Immunology, and Urology.

MRSA was detected from various specimens, including sputum, secretion, pus, urine, ascites, blood, tears, and others (Figure 3A). Among them, sputum yielded the most MRSA isolates (39.2%), followed by secretions (25.6%) and pus (14.8%). MRSA was also detected across multiple clinical departments (Figure 3B). The top five departments by MRSA detection rate were Respiratory Medicine (24.1%), General Surgery (14.7%), Dermatology (10.6%), Neurology (8.8%), and Orthopedics/Internal Medicine/Geriatrics (4.12% each).

**Figure 4:**
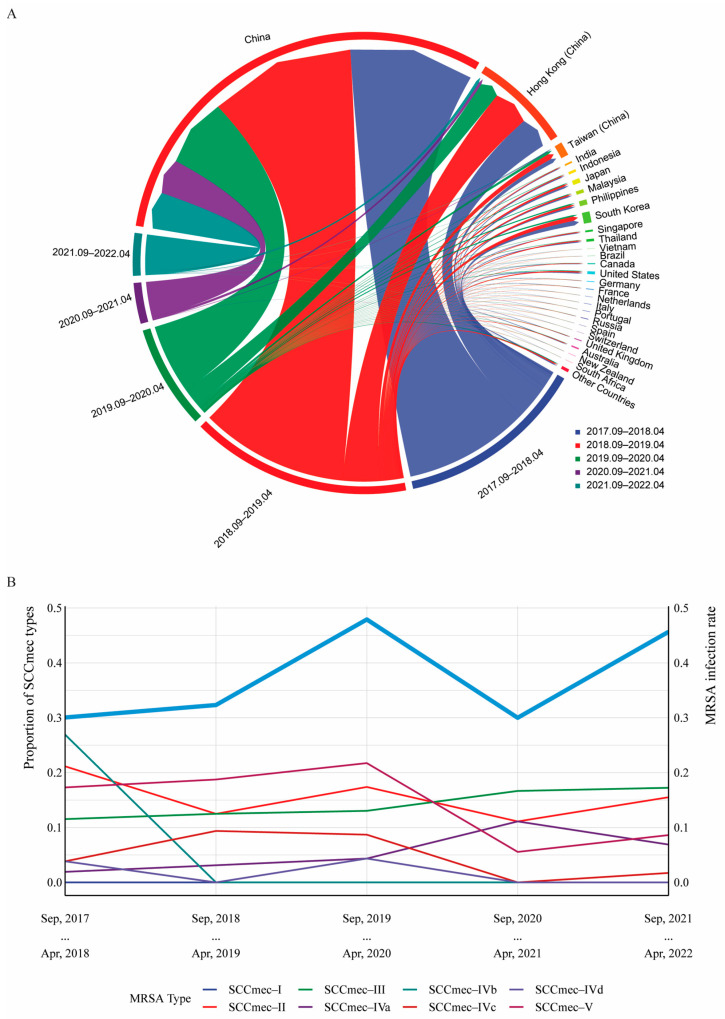
The correlations between MRSA occurrence and the annual number of travels in Macau (**A**). Inbound visitor numbers and distribution by country and region in Macau (Source: https://dataplus.Macautourism.gov.mo/, accessed on 21 October 2023). (**B**) The distribution trend of different *SCCmec* types. The thick blue line represents the change in MRSA rate over the years. Note: *SCCmec* IV (*SCCmec* IVa + *SCCmec* IVb + *SCCmec* IVc + *SCCmec* IVd).

**Figure 5:**
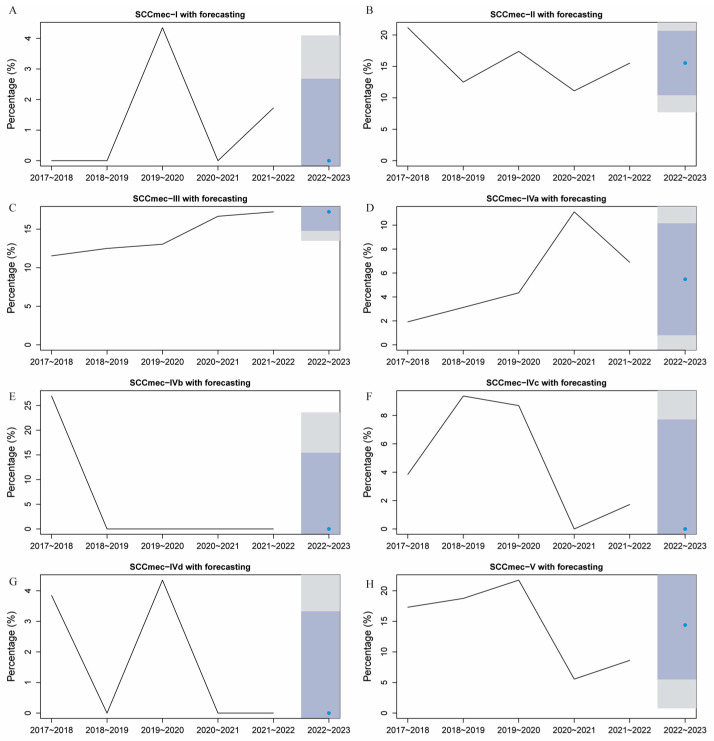
Prediction of MRSA phenotypes for the next year. (**A**) *SCCmec* I, (**B**) *SCCmec* II, (**C**) *SCCmec* III, (**D**) *SCCmec* IVa, (**E**) *SCCmec* IVb, (**F**) *SCCmec* IVc, (**G**) *SCCmec* IVd, (**H**) *SCCmec* V. Note: Circle indicate the predicted exact value. The gray and light purple bars represent the 95% and 80% confidence intervals, respecitively.

Having been a Portuguese colony for over 400 years, Macau has retained some European lifestyles. Simultaneously, it serves as an international tourist and gambling hub, attracting a multitude of visitors from various regions worldwide with over a million visitors annually (Figure 4A). Reflecting this, MRSA molecular epidemiology in Macau is diverse (Figure S2A–F). As depicted in Figure 4B, the detection rate of MRSA in Macau remained over 30% from 2017 to 2022, with a notable upward trend from 2017 to 2019. MRSA detection rates declined in 2020–2021, potentially due to reduced tourism during the COVID-19 pandemic. In the post-pandemic period of 2021–2022, MRSA detection rates in Macau rose again to 46%, suggesting a potential association between MRSA incidence and tourist numbers, though not statistically significant (*p* = 0.47). Distribution of MRSA phenotypes over time shows Types IV, II, III, and V as predominant in Macau, with Type III being most stable (Figure 4B). We further used ARIMA modeling to predict MRSA subtype distribution in Macau for 2022–2023 (Figure 5A–E). The forecasts show type III has maintained an increasing trend, and is expected to remain a predominant type in 2022–2023. Additionally, the predicted proportion of type II exceeds 10%, indicating that this MRSA type will also remain prevalent.

### 3.6. Drug Resistance Characteristics of MRSA in Macau

**Figure 6:**
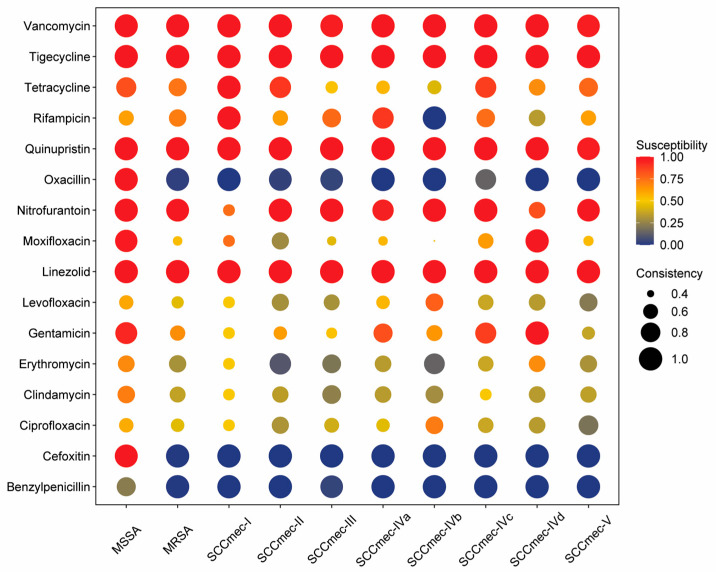
Antimicrobial susceptibility testing of MRSA and MSSA. Red indicates susceptibility to antimicrobials, while blue represents resistance. The size of the circle is used to assess the consistency of drug susceptibility across different MRSA subtypes and MSSA strains. A value of 1 signifies consistent susceptibility to the antimicrobial, with smaller values indicating poorer consistency, suggesting uncertainty in the resistance pattern of the samples to that particular antimicrobial. MRSA: Methicillin-resistant *Staphylococcus aureus*; MSSA: Methicillin-susceptible *Staphylococcus aureus*.

All 504 *S. aureus* isolates were examined for antimicrobial susceptibility; their anti-microbial resistance profiles are shown in Supplementary Table S3, and the relationship between MRSA *SCCmec* typing and drug resistance is shown in Figure 6. All *SCCmec* types I-V were resistant to Cefoxitin, Oxacillin, and Benzylpenicillin but sensitive to Vancomycin, Tigecycline, Quinupristin, Nitrofurantoin, and Linezolid. Resistance to partial antimicrobial agents was observed among the majority of isolates. Specifically, 69.4% of isolates demonstrated resistance to erythromycin. Clindamycin resistance was observed in 63.9% of isolates, while 51.7% showed resistance to levofloxacin, and 55.3% to ciprofloxacin. Resistance to moxifloxacin was demonstrated in 37.2% of isolates, and resistance to gentamicin and tetracycline was observed in 29.1% and 27.2%, respectively. Statistical tests revealed that resistance to Cefoxitin, Benzylpenicillin, Oxacillin, Gentamicin, Ciprofloxacin, Levofloxacin, Moxifloxacin, Erytromycin, and Clindamycin were significantly associated with MRSA infection (Table 4), with statistical significance (*p* < 0.05).

Comparison of antibiotic resistance between methicillin-susceptible and MRSA strains showed moxifloxacin (X-squared = 29.8, *p* = 0.0082), tetracycline (X-squared = 17.6, *p* = 0.0141), and rifampicin (X-squared = 29.9, *p* = 9.7 × 10^−5^) resistance were associated with MRSA infection.

## 4. Discussion

MRSA is a major cause of both hospital-acquired and community-acquired infections [35,36]. MRSA exhibits multidrug resistance, including resistance to β-lactam antibiotics, and high virulence, sometimes leading to fatal outcomes [37,38]. Due to its pathogenicity and pan-resistance, MRSA poses a considerable public health threat, especially in highly populated Macau with high volumes of international travel and a mixed Western and Eastern cultural heritage. To our best knowledge, this is the first comprehensive investigation of the uniqueness of MRSA in the clinical settings of Macau.

We employed a standardized annual sampling window from September through the following April for several reasons. First, winter and spring in Macau coincide with seasonal peaks in colds and influenza, corresponding to a heightened risk of hospital-acquired infections, as indicated by our preliminary data. Second, in Macau, tourism significantly fluctuates throughout the year, with peak seasons typically occurring in the months included in our collection period. The increase in tourist numbers may correlate with a rise in the spread of infectious diseases, including *S. aureus*, due to higher human traffic and density. This aspect was carefully considered, as it could introduce a variable that may affect the prevalence and diversity of the strains. Third, by maintaining a consistent collection window each year, we aimed to minimize variables that could potentially bias our data, thus ensuring comparability across the years within similar seasonal and tourist influx conditions. Finally, the sampling period has been approved by the hospital’s infection management department, with a focus on addressing the risks associated with peak tourist seasons.

The MRSA detection rate in Macau hospitals increased significantly from 2017 to 2022, rising from 30.0% to 47.9%. This surge surpassed the average MRSA detection rate in tertiary hospitals across Mainland China [14], indicating a notably higher prevalence of MRSA in the region and calling for heightened vigilance from the Macau Health Bureau. *SCCmec* is a mobile genetic element carrying the *mecA* gene, utilized for MRSA typing due to its essential components, including mec complexes, ccr complexes, and the J region. Types I, II, and III are predominantly associated with hospital-acquired MRSA (HA-MRSA), while types IV and V are commonly linked to community-acquired MRSA (CA-MRSA). This study, for the first time, conducted a study on the most common *SCCmec* types I to V in Macau’s MRSA strains. The findings indicated the detection of types II to V, with type IV being predominant, followed by types II, III, and V, excluding type I. This diversity in the *SCCmec* typing of Macau’s MRSA strains indirectly confirmed the coexistence of both CA-MRSA and HA-MRSA in the region. Previous data have indicated that the predominant strains in Asia are types II, III, and IV [6,14,39], and a multicenter longitudinal study in 2022 showed that epidemic typing of MRSA was ST59-t437-IV (14.9%), ST239-t030-III (6.4%) and ST5-t2460-II (6.0%) in Mainland China [40]. The diverse typing observed in Macau may be correlated with its geographical location. Furthermore, the influx of tourists from various parts of the world to Macau, including a significant number from Mainland China, Hong Kong, and Taiwan (data from dataplus.macaotourism.gov.mo), could contribute to this diversity. Projections from the ARIMA model suggest an upcoming decline in type IV prevalence, which might be associated with the prevalence of type II MRSA in the Guangdong province, a nearby region of Macau [40].

In terms of epidemiological characteristics, we found that age is positively associated with MRSA occurrence in Macau, with adults over 60 at a higher risk. Older adults tend to have weaker immunity and infection defenses [41]. Over 30% of MRSA cases originated from sputum, then by secretions and pus, aligning with prior reports [42,43]. This highlights the need to strengthen prevention, enhance sputum surveillance, and monitor bacterial strains in patients with lower respiratory tract infections.

Seasonal variation in *S. aureus* infections is controversial. Some studies have demonstrated surges in MRSA during warmer months and autumn in hospitals [44] and community [45] in temperate and tropical climates. However, we did not observe a significant correlation between seasonal fluctuations and MRSA infection in Macau. Several factors may account for this discrepancy. Macau’s unique subtropical climate with minimal temperature variations could mitigate any seasonal impact on MRSA. Increased international tourism and travel in Macau may introduce exogenous MRSA year-round, masking seasonal effects. Moreover, the persistent high density of the population in Macau could sustain endemic MRSA transmission, overriding seasonal factors. Further investigation is warranted to elucidate the reasons for the lack of seasonal variation in Macau’s distinctive setting. Elucidating the effects of climate, travel patterns, and population factors could better explain MRSA epidemiology in Macau and similar subtropical cities.

Additionally, we found universal susceptibility of Macau’s MRSA strains to five common antibiotics: vancomycin, tigecycline, quinupristin, nitrofurantoin, and linezolid (100% each). Prior studies also noted 100% MRSA susceptibility to both linezolid and quinupristin [46]. However, such broad universal susceptibility of MRSA to this panel of five antibiotics has not been previously documented.

Although our study provides an in-depth understanding of the trends and characteristics of MRSA in Macau, it is important to recognize certain limitations. The temporal scope (2017–2022) restricts a comprehensive grasp of long-term trends. Our ARIMA model predicted MRSA typing for the following year, but the unavailability of 2023 data (not yet disclosed by the Macau Health Bureau) hindered validation. Predominantly hospital-based samples may limit the generalizability of findings to community-acquired MRSA. Data constraints prevented exploration of influencing factors like patient travel histories and pathogen genetic variations. Future research should extend observation periods, include diverse community samples, and explore factors impacting MRSA epidemiology and antibiotic resistance in Macau’s subtropical urban settings.

## 5. Conclusions

In summary, our study highlights a relatively higher MRSA detection rate (30.0–47.9%) in Macau compared to mainland China and Europe over the last five years (2017–2022), with the *SCCmec* type IV strain predominantly present, followed by types II, III, and V. Projections from the ARIMA model suggest an upcoming decline in type IV prevalence, accompanied by an increase in types II and III, indicating that the clone with *SCCmec* IV is probably correlated with the fitness of the clones with *SCCmec* II and III. Notably, despite seasonal fluctuations and international travel, hospital detection rates remained consistent. Moreover, sputum, secretions, and pus emerged as the most frequently detected specimens, with the respiratory department observing the highest MRSA incidence. Our findings underscore advanced age as a susceptibility factor for MRSA infections. Furthermore, our investigation revealed diverse antibiotic resistance profiles among MRSA strains in Macau, demonstrating universal sensitivity to vancomycin, tigecycline, quinupristin, nitrofurantoin, and linezolid, alongside complete resistance to cefoxitin, oxacillin, and benzylpenicillin. This comprehensive analysis highlights the unique epidemiological and resistance patterns of MRSA in Macau, emphasizing the significance of tailored, proactive measures for the effective management and control of this pathogen within the region.

## Figures and Tables

**Table 1:** Allelic profile of each *S. aureus* isolate.

Isolate	arcC	aroE	glpF	gmk	pta	tpi	yqiL	ST	Clonal Complex
S1_MSSA	13	13	1	1	12	11	13	15	CC15
S2_MSSA	2	2	2	2	6	3	2	30	CC30
S3_ATCC25923	1	4	1	4	12	1	10	5	CC5
S4_MRSA	19	23	15	2	19	20	15	59	
S5_MRSA	10	14	8	6	10	3	2	45	CC45
S6_MSSA	2	2	2	2	6	3	2	30	CC30
S7_MSSA	5	4	1	4	4	6	3	7	
S8_MRSA	2	2	2	2	6	3	2	30	CC30
S9_MSSA	3	35	19	2	20	26	39	398	
S10_MRSA	10	14	8	6	10	3	2	45	CC45
S11_MRSA	10	14	8	6	10	3	2	45	CC45
S12_MRSA	10	14	8	6	10	3	2	45	CC45

Note: MRSA: Methicillin-resistant *Staphylococcus aureus*; MSSA: Methicillin-susceptible *Staphylococcus aureus*; ST: sequence type; CC: clonal complex.

**Table 2:** Clinical characteristics of subjects stratified by methicillin susceptibility.

Patient Characteristics	MSSA (N = 321)	MRSA (N = 183)	*p*
	63.7%	36.3%	
Period (%)			
2017.09–2018.04	121 (37.7)	52 (28.4)	0.018
2018.09–2019.04	64 (19.9)	32 (17.5)	
2019.09–2020.04	25 (7.8)	23 (12.6)	
2020.09–2021.04	42 (13.1)	18 (9.8)	
2021.09–2022.04	69 (21.5)	58 (31.7)	
Season (%)			
Spring	151 (54.9)	81 (47.1)	0.131
Winter	124 (45.1)	91 (52.9)	
Gender (%)			
Female	116 (41.9)	68 (37.2)	0.361
Male	161 (58.1)	115 (62.8)	
Age (mean ± SD)	47.2 (31.9)	62.2 (28.4)	<0.001
Age (Group) (%)			
[0, 1)	41 (14.8)	9 (4.9)	<0.001
[1, 6)	17 (6.1)	6 (3.3)	
[13, 18)	5 (1.8)	1 (0.5)	
[18, 40)	42 (15.2)	23 (12.6)	
[30, 60)	45 (16.2)	22 (12.0)	
[6, 13)	7 (2.5)	3 (1.6)	
[60, 80)	62 (22.4)	46 (25.1)	
[80, -)	58 (20.9)	73 (39.9)	
Inpatient (%)			
Inpatient	158 (64.0)	122 (76.7)	0.009
Outpatient	89 (36.0)	37 (23.3)	

Data are mean ± standard deviation (SD) or median and interquartile range (IRQ) depending on the distribution. Differences between MSSA and MRSA were analyzed using either Student’s t-tests (continuous data) or Mann–Whitney U tests (nonparametric continuous data) or Chi-square tests (categorical data). The proportions between the two groups were analyzed using the two-proportion z-test. *p* < 0.05 was statistically significant. MRSA: Methicillin-resistant *Staphylococcus aureus*; MSSA: Methicillin-susceptible *Staphylococcus aureus*.

**Table 3:** The patient characteristics stratified by inpatient or outpatient status.

MRSA Type		Inpatient	Outpatient	*p*
		(N = 280)	(N = 126)	
		69.0%	31.0%	
*SCCmec*-I (%)	Negative	279 (99.6)	126 (100.0)	1
	Positive	1 (0.4)	0 (0.0)	
*SCCmec*-II (%)	Negative	262 (93.6)	119 (94.4)	0.908
	Positive	18 (6.4)	7 (5.6)	
*SCCmec*-III (%)	Negative	255 (91.1)	123 (97.6)	0.028
	Positive	25 (8.9)	3 (2.4)	
*SCCmec*-IV (%)	Negative	259 (92.5)	117 (92.9)	1
	Positive	21 (7.5)	9 (7.1)	
*SCCmec*-V (%)	Negative	260 (92.9)	123 (97.6)	0.091
	Positive	20 (7.1)	3 (2.4)	

Differences between inpatients and outpatients were analyzed using Chi-square tests (categorical data). The proportions between the two groups were analyzed using the two-proportion z-test. *p* < 0.05 was statistically significant.

**Table 4:** Antimicrobial susceptibility testing of *S. aureus* and MRSA.

Antimicrobial		*S. aureus* (SA) (N = 321)	MRSA (N = 183)	*p*
Cefoxitin (%)	Resistant	6 (1.9)	179 (99.4)	<0.001
	Sensitive	313 (98.1)	1 (0.6)	
Benzylpenicillin (%)	Resistant	247 (77.4)	178 (99.4)	<0.001
	Sensitive	72 (22.6)	1 (0.6)	
Oxacillin (%)	Resistant	5 (1.6)	175 (97.8)	<0.001
	Sensitive	314 (98.4)	4 (2.2)	
Gentamicin (%)	Intermedia	7 (2.2)	17 (9.5)	<0.001
	Resistant	18 (5.6)	52 (29.1)	
	Sensitive	294 (92.2)	110 (61.5)	
Ciprofloxacin (%)	Resistant	132 (41.4)	99 (55.3)	0.004
	Sensitive	187 (58.6)	80 (44.7)	
Levofloxacin (%)	Intermedia	11 (3.4)	14 (7.8)	<0.001
	Resistant	123 (38.6)	93 (51.7)	
	Sensitive	185 (58.0)	73 (40.6)	
Moxifloxacin (%)	Intermedia	10 (3.1)	33 (18.3)	<0.001
	Resistant	7 (2.2)	67 (37.2)	
	Sensitive	302 (94.7)	80 (44.4)	
Erythromycin (%)	Intermedia	1 (0.3)	2 (1.1)	<0.001
	Resistant	103 (32.4)	125 (69.4)	
	Sensitive	214 (67.3)	53 (29.4)	
Clindamycin (%)	Intermedia	0 (0.0)	1 (0.6)	<0.001
	Resistant	94 (29.5)	115 (63.9)	
	Sensitive	225 (70.5)	64 (35.6)	
Quinupristin (%)	Resistant	2 (0.6)	1 (0.6)	1.000
	Sensitive	317 (99.4)	179 (99.4)	
Linezolid (%)	Resistant	1 (0.3)	0 (0.0)	1.000
	Sensitive	317 (99.7)	179 (100.0)	
Vancomycin (%)	Resistant	4 (1.3)	3 (1.7)	1.000
	Sensitive	315 (98.7)	177 (98.3)	
Tetracycline (%)	Intermedia	1 (0.3)	0 (0.0)	0.020
	Resistant	54 (17.0)	49 (27.2)	
	Sensitive	263 (82.7)	131 (72.8)	
Tigecycline (%)	Resistant	1 (0.3)	0 (0.0)	1.000
	Sensitive	318 (99.7)	179 (100.0)	
Nitrofurantoin (%)	Intermedia	2 (0.6)	5 (2.8)	0.111
	Resistant	1 (0.3)	0 (0.0)	
	Sensitive	316 (99.1)	175 (97.2)	
Rifampicin (%)	Intermedia	3 (0.9)	2 (1.1)	0.125
	Resistant	121 (37.9)	52 (28.9)	
	Sensitive	195 (61.1)	126 (70.0)	

Note: The proportions between the two groups were analyzed using the two-proportion z-test. *p* < 0.05 was statistically significant.

## Data Availability

All the data relevant to this manuscript are available upon request from the corresponding author.

## Supplementary Materials

The following supporting information can be downloaded at: https://www.mdpi.com/article/10.3390/microorganisms12010148/s1. Table S1: Primer sequences and amplification conditions of all genes, Table S2: Antimicrobial susceptibility and their antimicrobial resistance profiles based on Clinical and Laboratory Standards Institute (CLSI) guidelines (2017); Figure S1: The identification of MRSA strains in Macau (A) Electrophoretic analysis of the amplified fragments by using the multiplex PCR in 1.2% agarose gel stained with ethidium bromide. Lane M: molecular weight marker (100-bp DNA Ladder); Lane 1: *SCCmec*-I (613-bp); Lane 2: *SCCmec*-II (398-bp); Lane 3: *SCCmec*-III (280-bp); Lane 4: *SCCmec*-IVa (776-bp); Lane 5: *SCCmec*-IVb (493-bp); Lane 6: *SCCmec*-IVc (200-bp); Lane 7: *SCCmec*-IVd (881-bp); Lane 8: *SCCmec*-V (325-bp). (B) Phylogenetic tree of the 8 *SCCmec* genes used in this study. (C) Correlation heatmap of sequences, where red indicates higher correlation and blue indicates lower correlation. (D) Multiple alignment of selected gene sequences; Figure S2: The distribution of different *SCCmec* types and subtypes. (A). Sep, 2017 to Apr, 2018; (B) Sep, 2018 to Apr, 2019; (C) Sep, 2019 to Apr, 2020; (D) Sep, 2020 to Apr, 2021; (E) Sep, 2021 to Apr, 2022; (F) All. Note: I: *SCCmec* I; II: *SCCmec* II; III: *SCCmec* III; IVa: *SCCmec* IVa; IVb: *SCCmec* IVb; IVc: *SCCmec* IVc; IVd: *SCCmec* IVd; V: *SCCmec* V; *SCCmec* IV (*SCCmec* IVa + *SCCmec* IVb + *SCCmec* IVc + *SCCmec* IVd).
