# Supplementary material for: The Prevalence, Epidemiological, and Molecular Characterization of Methicillin-Resistant Staphylococcus aureus (MRSA) in Macau (2017–2022)
Source: Microorganisms. 2024 Jan 12;12(1):148. doi: 10.3390/microorganisms12010148 (PMC10820975; doi:10.3390/microorganisms12010148)
Supplement: Supplementary file 1 [file microorganisms-12-00148-s001.zip › Supplemental materials_2023-12-16-final.pdf]

Supplementary Material

The Prevalence, Epidemiological and Molecular Characterization of Methicillin-resistant *Staphylococcus aureus* (MRSA) in Macau (2017 – 2022)

Abao Xing[1 †], HoiMan Ng[2†], Huining Jiao[3], Kefeng Li[1] and Qianhong Ye[3]*

1 Faculty of Applied Sciences, Macao Polytechnic University, Macau; p2314405@mpu.edu.mo (A.X.), kefengl@mpu.edu.mo (K.L.)

2 Clinical laboratory, Kiang Wu Hospital, Macau; alvin_ng@163.com

3 Faculty of Health Sciences and Sports, Macao Polytechnic University, Macau; p2010371@mpu.edu.mo (H.J.); yeqianhong@mpu.edu.mo (Q.Y.)

*Authors to whom correspondence should be addressed: yeqianhong@mpu.edu.mo

†Contributed equally to this work as co-first authors (A.X. and H.N.).

1. Supplementary Method

Sample collection

Samples were typically obtained using sterile swabs or other appropriate collection tools from sites relevant to *Staphylococcus aureus* (SA) colonization or infection, such as nasal passages, wounds, or skin lesions. Conditions during sample collection were maintained as sterile as possible to minimize the risk of contamination. Healthcare professionals need some precautions, including wearing gloves and adopting proper hand hygiene practices, to prevent cross-contamination between samples and to safeguard against potential infection spread.

Proper labeling and handling of specimens were ensured to maintain sample integrity. Adequate documentation of patient information, including medical history and antibiotic usage, was considered to provide a comprehensive context for the analysis. To mitigate the risk of contamination, stringent laboratory practices were followed (See details of operations in follow chapters). Workspaces and equipment were regularly sanitized, and separate areas were designated for different steps of the analysis to prevent cross-contamination.

Strain isolation

The strains and standard freeze-dried strains were extracted using a triangular cotton swab. The strains were streaked onto blood agar plates and cultured at 37℃ for 18-24 hours. Subsequently, the obtained colonies were inoculated onto trypticase soy agar (TSA) medium and cultured at 37℃ for 18-24 hours, resulting in the second-generation strains. These second-generation strains were then inoculated onto TSA medium and cultured at 37℃ for 18-24 hours to obtain the third-generation strains.

Colonies selected from TSA were added to physiological saline, and a homogeneous 0.5 McFarland turbidity bacterial suspension was prepared. Using a cotton swab, an appropriate amount of bacterial suspension was evenly spread on Mueller-Hinton (MH) agar. After complete absorption of the liquid, antimicrobial susceptibility paper disks were applied, and the plates were cultured at 37℃ for 24 hours. The interpretation was performed based on the antimicrobial susceptibility testing interpretive criteria according to the standards based on the 2017 Clinical and Laboratory Standards Institute (CLSI) guidelines, with the inhibition zone diameter measured using a ruler.

Sample preparation for VITEK-2

Before usage, retrieve the cards (made in the strain isolation step) and saline bottles from the refrigerator and allow them to equilibrate at room temperature (about 20-25℃) for 15-20 minutes to ensure thorough warming. Place disposable plastic tubes on the card rack, adding 3 mL of 0.45% NaCl solution to each tube. Calibrate the turbidimeter using the calibration tube, ensuring that the measured values fall within the specified range. Depending on requirements, prepare a bacterial suspension, and measure the suspension concentration using the turbidimeter. In the case of concurrent antimicrobial susceptibility testing, dilute and mix the suspension as needed.

Antimicrobial susceptibility testing

The determination test for MRSA is conducted according to the standards of the Clinical and Laboratory Standards Institute (CLSI) using the Kirby-Bauer (K-B) disk diffusion method with cefoxitin (30μg/disk). The criteria for interpretation are as follows: an inhibition zone diameter ≦ 21mm indicates MRSA, while an inhibition zone diameter ≧22mm indicates Meticillin-Sensitive *Staphylococcus aureus* (MSSA).

For preparation, bacterial colonies from trypticase soy agar (TSA) were selected, mixed with 3mL of physiological saline, and adjusted to a 0.5 McFarland turbidity standard. Sterile cotton swabs were used to apply the bacterial suspension evenly on Mueller-Hinton agar. After complete absorption of the bacterial suspension, antibiotic disks were placed on the agar. The application process, involving 5 to 6 disks per agar plate, was completed within 15 minutes. The distance between disks was no less than 24mm, and the distance from the center of the disk to the edge of the agar plate was not less than 15mm. After application, the plates were inverted and incubated at 37°C for 24 hours before interpretation.

Interpretation of results followed CLSI guidelines for *Staphylococcus* antimicrobial susceptibility testing. Measurements were taken using a ruler to determine the inhibition zone diameter, considering the point where the bacteria were not visible from the center to the edge of the disk. Quality control involved using methicillin-sensitive strain ATCC25923 and methicillin-resistant strain ATCC700699, with inhibition zone diameters of 17/27 within permissible ranges for accurate reporting.

DNA preparation

For DNA extraction, overnight cultured isolates were inoculated into 2 mL Luria-Bertani (LB) medium and incubated at 37 ℃ for 180 rpm until reaching an OD600 of 0.8-1. Bacterial DNA was extracted using the Rapid Bacterial Genomic DNA Isolation Kite (Sangon Biotech, Shanghai, China) according to the manufacturer ’ s instructions. Extracted DNA was quantified using Nanodrop ND-1000 (Thermo Fisher Scientific, USA) and stored at -20 ℃ for further analysis.

2. Supplementary Figures

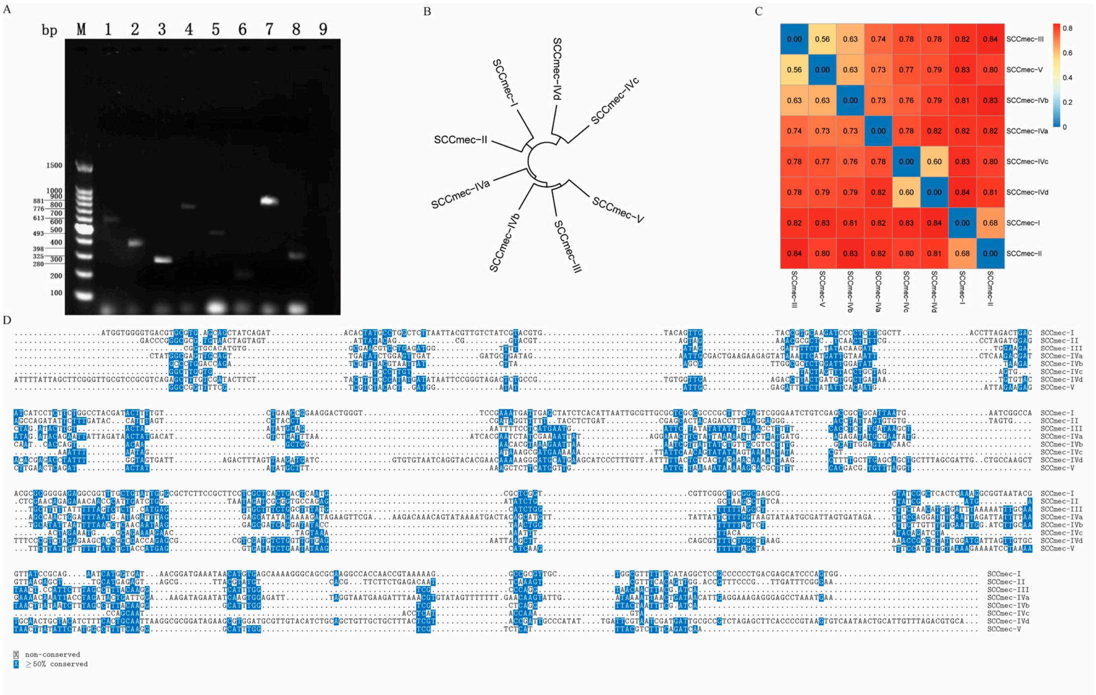

Figure S1. The identification of MRSA strains in Macau (A) Electrophoretic analysis of the amplified fragments by using the multiplex PCR in 1.2% agarose gel stained with ethidium bromide. Lane M: molecular weight marker (100-bp DNA Ladder); Lane 1: *SCCmec*-I (613-bp); Lane 2: *SCCmec*-II (398-bp); Lane 3: *SCCmec*-III (280-bp); Lane 4: *SCCmec*-IVa (776-bp); Lane 5: *SCCmec*-IVb (493-bp); Lane 6: *SCCmec*-IVc (200-bp); Lane 7: *SCCmec*-IVd (881-bp); Lane 8: *SCCmec*-V (325-bp). (B) Phylogenetic tree of the 8 *SCCmec* genes used in this study. (C) Correlation heatmap of sequences, where red indicates higher correlation and blue indicates lower correlation. (D) Multiple alignment of selected gene sequences.

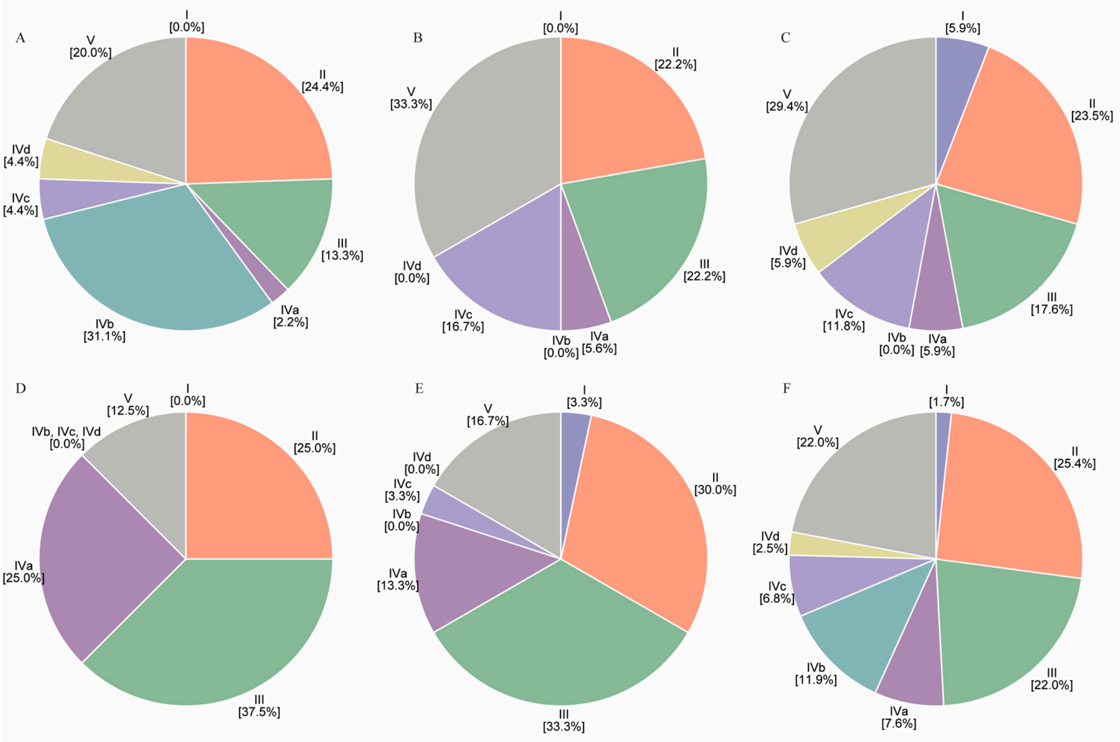

Figure S2. The distribution of different *SCCmec* types and subtypes. (A). Sep, 2017 to Apr, 2018; (B) Sep, 2018 to Apr, 2019; (C) Sep, 2019 to Apr, 2020; (D) Sep, 2020 to Apr, 2021; (E) Sep, 2021 to Apr, 2022; (F) All. Note: I: *SCCmec* I; II: *SCCmec* II; III: *SCCmec* III; IVa: *SCCmec* IVa; IVb: *SCCmec* IVb; IVc: *SCCmec* IVc; IVd: *SCCmec* IVd; V: *SCCmec* V; *SCCmec* IV (*SCCmec* IVa + *SCCmec* IVb + *SCCmec* IVc + *SCCmec* IVd).
